# Genetic insights into the gut microbiota, herpes zoster, and postherpetic neuralgia: a bidirectional two-sample Mendelian randomization study

**DOI:** 10.3389/fgene.2024.1366824

**Published:** 2024-05-23

**Authors:** Zhimin Deng, Yali Liu, Haiying Wang, Tianyuan Luo

**Affiliations:** ^1^ Department of Anesthesiology, Affiliated Hospital of Zunyi Medical University, Zunyi, China; ^2^ Guizhou Key Laboratory of Anesthesia and Organ Protection, Zunyi, China

**Keywords:** Mendelian randomization, gut microbiota, herpes zoster, postherpetic neuralgia containing, causal effect

## Abstract

**Background:**

An increasing amount of evidence suggests that gastrointestinal diseases are risk factors for herpes zoster (HZ) and postherpetic neuralgia (PHN). Among them, the gut microbiota may play a crucial role in this process. Therefore, this study aims to explore the potential causal association between the gut microbiota and HZ and PHN.

**Methods:**

Bidirectional two-sample Mendelian randomization (MR) analysis was used to detect the causal effect between HZ and PHN and the gut microbiota. Gut microbiota data were derived from the MiBioGen consortium, while HZ and PHN data were obtained from the FinnGen database. We selected single-nucleotide polymorphisms (SNPs) as instrumental variables with a threshold of *p* < 1 × 10⁻⁵ for the association with the gut microbiota in forward MR analysis and *p* < 5 × 10^⁻8^ for the association with HZ or PHN in reverse MR analysis and then removed SNPs in linkage disequilibrium (*r* ^2^ < 0.001) within a distance of 10,000 kb for both the gut microbiota and HZ and PHN. These SNPs were utilized to assess the causal effect between exposures and outcomes using inverse-variance weighting (IVW), MR–Egger, weighted mean, and weighted median tests.

**Results:**

The class Deltaproteobacteria, order Desulfovibrionales, family Desulfovibrionaceae, and genus *Coprococcus 2* were found to reduce the risk of HZ, while the phylum Cyanobacteria, genus *Eubacterium rectale* group appeared to increase it. The class Coriobacteriia, order Coriobacteriales, family Coriobacteriaceae, genus *Lachnospiraceae NK4A136* and genus *Ruminococcaceae UCG011* were found to reduce the risk of PHN, while the genus *Candidatus Soleaferrea*, genus *Eubacterium rectale* group, and genus *Methanobrevibacter* appeared to increase it. Moreover, the onset of HZ was found to increase the level of the genus *Eubacterium rectale* group. These findings remained robust and unaffected by heterogeneity or horizontal pleiotropy among SNPs in both forward and reverse MR analysis.

**Conclusion:**

This MR study provided evidence supporting a potential causal relationship between the gut microbiota and HZ and PHN. Moreover, we found that the causal effect between the gut microbiota and HZ is bidirectional. Further studies are required to clarify the biological mechanisms linking the gut microbiota and these conditions.

## 1 Introduction

Herpes zoster (HZ) is a common viral infection that arises from the reactivation of the varicella zoster virus (Kim et al., 2021), which is known to remain dormant in the dorsal root ganglion (De Oliveira Gomes et al., 2023). The disease is characterized by the intense inflammation of the affected nerves and skin with a distribution of painful grouped vesicles on an erythematous base (Zhang J. et al., 2023). A population-based study conducted in the United States showed that over 65% of adult patients with HZ were provided with pain medication to alleviate acute herpetic pain (Lin et al., 2019). Complications are frequently encountered, and research indicates that postherpetic neuralgia (PHN) remains a prominent clinical issue in China (Wen et al., 2023), with a prevalence of up to 29.8% among patients experiencing ongoing neuropathic pain following acute HZ reactivation (Sim et al., 2021). PHN is considered a common, serious, and painful complication of HZ (Jiang X. et al., 2023), which substantially lowers the patient’s quality of life and often requires medical intervention (Lu et al., 2023). Although extensive research shows that many risk factors such as aging, hypoimmunity, initial infection severity, and medical conditions are modifiable, the management of patients with HZ and PHN remains challenging.

The gut microbiota, i.e., a large number of complex microorganisms in the body gut, has a significant correlation with the development of type 2 diabetes (Liu et al., 2023), autoimmune diseases (Shao et al., 2023), obesity (Duan et al., 2023), and other multiple diseases. Additionally, the complex gut microbiota plays a pivotal role in a wide range of essential physiological processes, encompassing and safeguarding the body against pathogen invasion, fostering the development of a resilient immune system, aiding in digestion and the absorption of nutrients, supporting body growth and metabolic functions, and fortifying immune responses against tumors (Kurilshikov et al., 2021; Zhang F. et al., 2023). At present, there are few reports and studies on the gut microbiota and HZ or PHN at home and abroad. However, there is evidence indicating that in inflammatory bowel disease, a disease closely related to intestinal microbiota disturbance, the incidence of HZ was higher than that of non-inflammatory bowel disease (Côté-Daigneault et al., 2019). A study shows that ulcerative colitis and Crohn’s disease patients had a 1.35- and 1.66-fold increased infection risk of HZ, respectively, compared with controls (Singer et al., 2023). As mentioned above, the gut microbiota is involved in the regulation of immunity, and the low function of immunity is the main reason for the reactivation of the varicella zoster virus and the development of HZ and PHN. Thus, it is crucial to elucidate the causal relationship between the gut microbiota and HZ PHN.

Different from randomized controlled clinical trials, Mendelian randomization (MR) is an alternative and effective method that uses single-nucleotide polymorphisms (SNPs) as instrumental variables (IVs) to infer the causation between exposure and outcome (Clayton et al., 2023). Nonetheless, in certain scenarios, there might be a reverse causation where the outcome variable affects the exposure variable as well. Bidirectional MR analysis addresses this by examining the causal effects in both directions. By comparing the results of both analyses, we can ascertain whether a causal relationship exists between the exposure and outcome variables or if reverse causation exists. Hence, in this study, we performed a bidirectional two-sample MR analysis utilizing genome-wide association study (GWAS) data to uncover causal associations between the gut microbiota and HZ and PHN. The findings of this study may provide novel evidence linking the gut microbiota to HZ and PHN.

## 2 Methods

**FIGURE 1:**
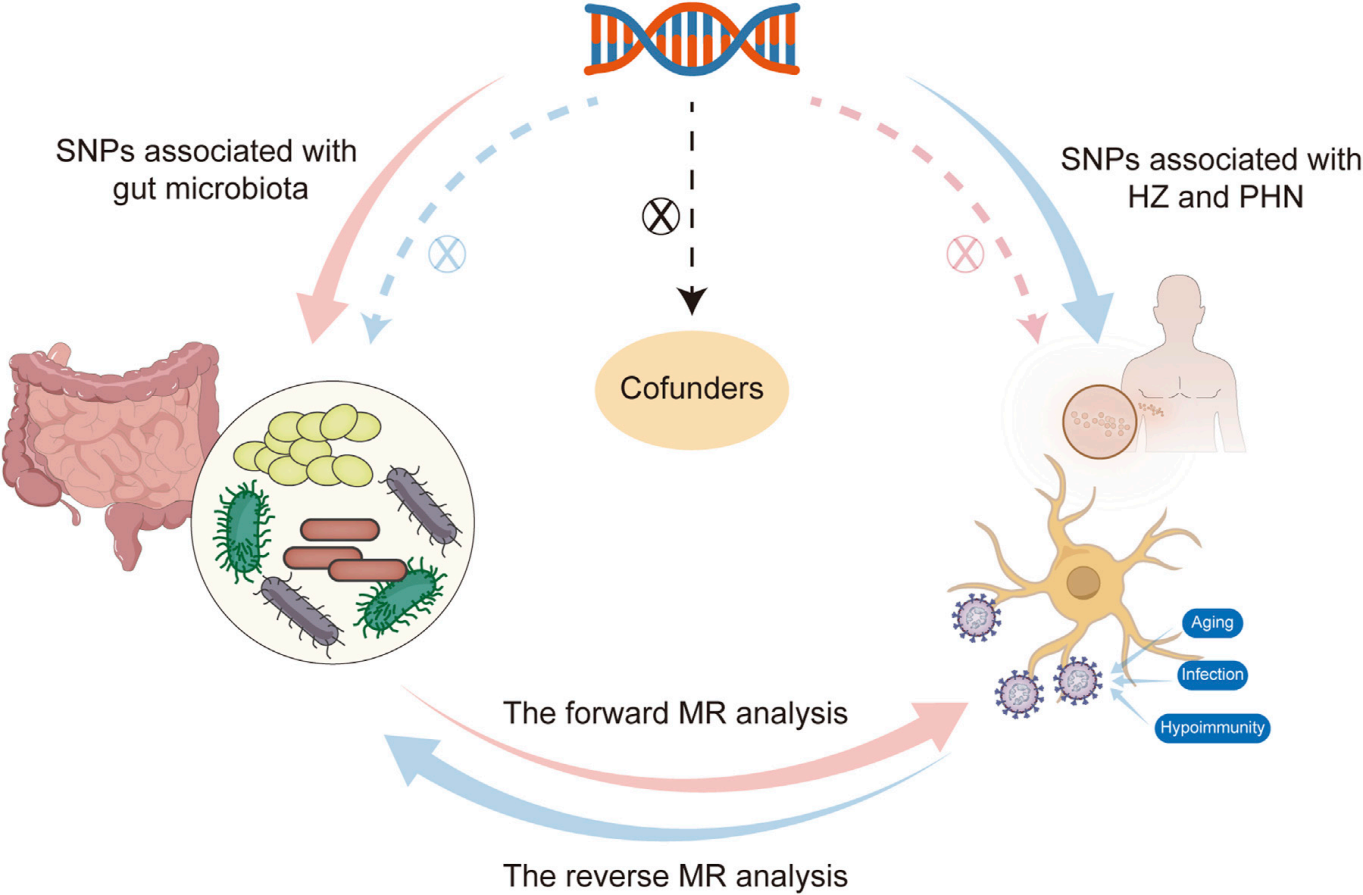
Flowchart of this bidirectional Mendelian randomization (MR) study 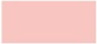
shows that the forward MR analysis takes the gut microbiota as the exposure factor and HZ and PHN as the outcome variable, and the dashed line indicates that single-nucleotide polymorphisms (SNPs) are not associated with outcomes. 
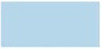
shows that the reverse MR analysis takes HZ and PHN as the exposure variable and the significant microorganisms that are associated with the occurrence of HZ and PHN in forward MR analysis as the outcome variable, and the dashed line indicates that SNPs are not associated with outcomes. 
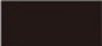
shows that SNPs are not associated with confounding factors. HZ, herpes zoster; MR, Mendelian randomization; PHN, postherpetic neuralgia.

Traditional MR analysis primarily focuses on the causal relationship in a single direction, whereas bidirectional MR analysis further considers the bidirectional causal relationship between exposure and outcome. Bidirectional MR analysis provides a more thorough and accurate assessment of the causal link between the exposure variable and outcome variable. Hence, we first selected the gut microbiota as the exposure variable and HZ and PHN as the outcome variable to detect whether the gut microbiota promotes or prevents the occurrence of HZ and PHN. Then, we also investigated changes in the gut microbiome after the occurrence of HZ and PHN in the reverse MR analysis. The selection of these genetic IVs for phenotypes adhered to three primary assumptions: 1) the IVs should exhibit a strong association with their corresponding phenotype; 2) the IVs should remain unaffected by potential confounding factors that could influence the relationship between the exposure and outcome; and 3) there should be no direct links between the IVs and the outcome (Figure 1). Ethical approval was not required for our analysis as we utilized publicly available GWAS results from the FinnGen database and the MiBioGen consortium.

### 2.1 Data source and SNP selection

The gut microbiota were composed of 211 taxa, including 9 phyla, 16 classes, 20 orders, 35 families, and 131 genera. Summary statistics for gut microbiota taxa were obtained from the largest available GWAS meta-analysis conducted by the MiBioGen consortium, after adjusting for age, gender, technical variables, and genetic principal components (Ye et al., 2023), including 18,340 individuals of 16S rRNA genes from 24 cohorts of European, African, Asian, Middle Eastern, and Hispanic ancestry (Kurilshikov et al., 2021). Summary statistics for HZ and PHN were both from a European population and from the FinnGen database round 9. For HZ, the total number of participants was 367,970, with 4,973 cases and 362,997 controls, including 20,170,065 SNPs. For PHN, the total number of participants was 330,690, with 313 cases and 330,377 controls, including 20,169,134 SNPs (Table 1). In sample-wise quality control steps of the FinnGen database, individuals with ambiguous gender, high genotype missingness (>5%), excess heterozygosity (+−4SD), and non-Finnish ancestry were excluded. In variant-wise quality control steps, variants with high missingness (2%), low Hardy-Weinberg equilibrium (HWE) *p*-value (<1e-6), and low allele count (MAC<3) were excluded. There was no confounding effect in the samples between these datasets.

**TABLE 1:** Description of the GWAS summary statistics of the gut microbiota, herpes zoster, and postherpetic neuralgia.

Trait	Sample size (case)	Number of SNPs	Population	Reference
Gut microbiota	18,340	5,717,754	Mixed	PM ID: 33462485
Herpes zoster	4,973 vs*.* 362,997	20,170,065	European	—
Postherpetic neuralgia	313 vs*.* 330,377	20,169,134	European	—

We utilized publicly available GWAS results of herpes zoster and postherpetic neuralgia from the FinnGen database and the gut microbiota data from the MiBioGen consortium.

GWAS, genome-wide association study.

For our genome-wide analysis, we employed representative SNPs as genetic IVs with a threshold of *p* < 1 × 10⁻⁵ for the association with the gut microbiota in the forward MR analysis and *p* < 5 × 10^⁻8^ for the association with HZ or PHN in the reverse MR analysis. To ensure the independence of SNPs, we used the clump data function within the TwoSampleMR package to remove SNPs in linkage disequilibrium (*r* ^2^ < 0.001) within a distance of 10,000 kb (Zhong et al., 2023a).

### 2.2 Statistical analysis

In this study, the inverse-variance weighting (IVW) method, MR–Egger, weighted mean, and weighted median tests were used for Mendelian randomization analysis (Thorkildsen et al., 2023). We employed the IVW method as the main analysis approach to examine the causal effect (Ye et al., 2023). The strength of the SNPs used as the instrument was assessed using the *F*-statistic (*F* = beta^2^/Se^2^) (Jiang H. et al., 2023; Shi et al., 2023). We only considered SNPs with an *F*-statistic exceeding 10 in order to mitigate potential biases stemming from weak instruments (Huang et al., 2021).

The exposure and outcome data in the two-sample MR analysis came from different samples, and there may be population differences, so it is necessary to conduct the heterogeneity test. In this study, heterogeneity was examined using the MR–Egger and IVW tests in Cochran’s Q statistic. An observed *p* value > 0.05 suggests the non-existence of heterogeneity. From a statistical perspective, horizontal pleiotropy means that an SNP known to influence the hypothesized exposure also influences the hypothesized outcome by other pathways. In this study, horizontal pleiotropy was assessed using MR–Egger intercept analysis; if there was significant pleiotropy between SNPs, they would be further analyzed with outlier-corrected MR-PRESSO. The MR analysis was re-conducted after removing the outliers (Rodríguez-Fernández et al., 2022). Furthermore, we conducted a leave-one-out sensitivity analysis to show that the causal effect of exposure on the outcome remained unaffected by individual SNPs. All analyses were performed using the TwoSampleMR package (version 0.5.7), Mendelian Randomization package (version 0.8.0), and MR-PRESSO package (version 1.0.0) in R software 4.3.1 (https://www.R-project.org). A statistical significance level of *p* < 0.05 was adopted to determine whether there was any evidence of a potential causal effect.

## 3 Results

### 3.1 Causal effects of the gut microbiota on HZ

**FIGURE 2:**
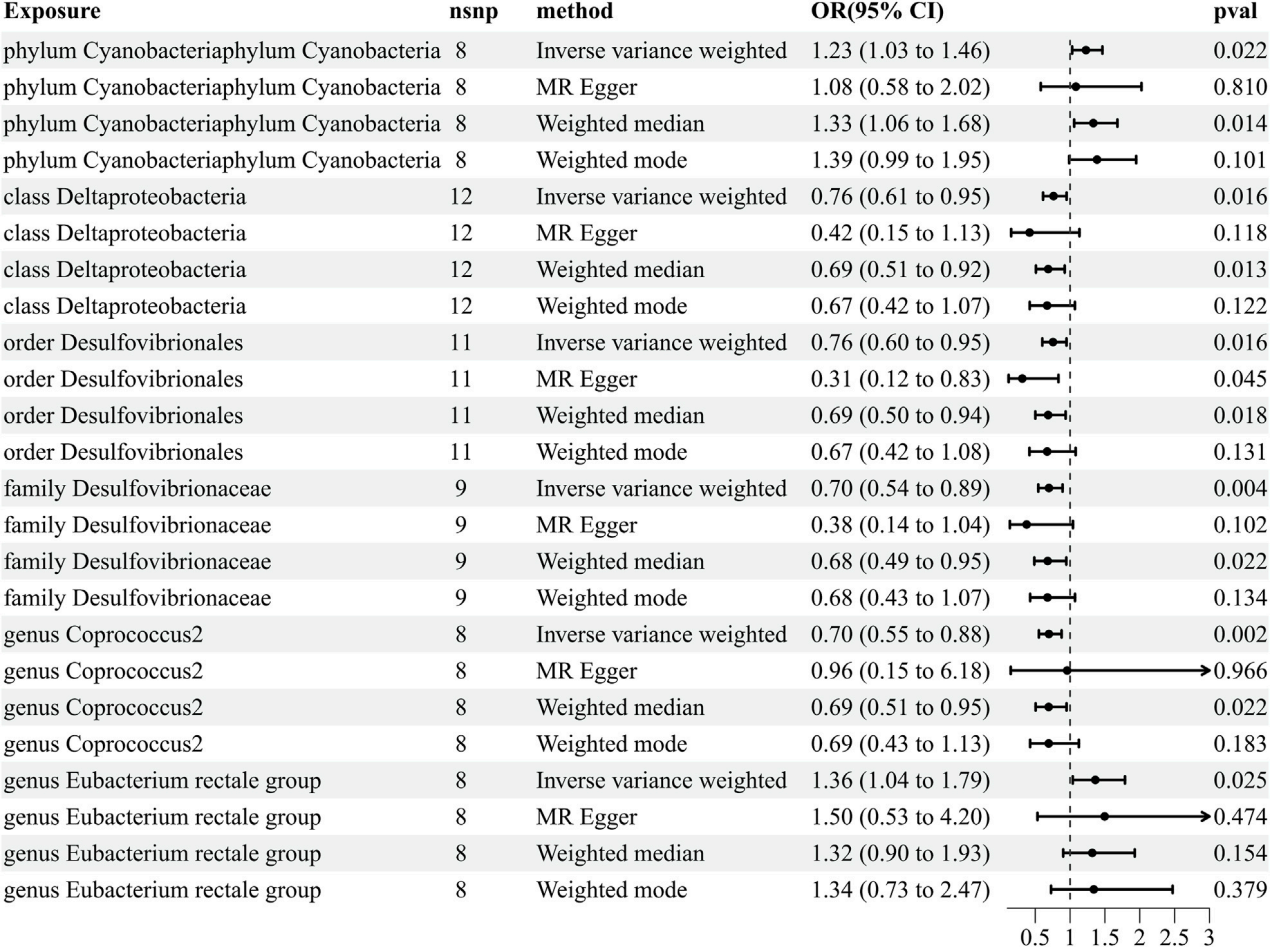
Results of MR estimating the causal association between the gut microbiota and herpes zoster. The forward MR analysis uses the gut microbiota as exposure variable and herpes zoster risk as the outcome variable, and using the IVW method as the main result, effect sizes with 95% confidence intervals, the number of SNPs, and *p*-values were presented. CI, confidence interval; HZ, herpes zoster; IVW, inverse-variance weighted; MR, Mendelian randomization; OR, odds ratio.

Our comprehensive MR analysis revealed that six bacterial traits exhibited statistically significant associations with HZ. These IVs were found in a diverse set of taxa, including 1 phylum (with 8 SNPs), 1 class (with 12 SNPs), 1 order (with 11 SNPs), 1 family (with nine SNPs), and 2 genera (with 16 SNPs). Notably, each SNP demonstrated adequate validity, with all *F-*values greater than 10. The IVW results showed that four bacterial genera were protective against HZ, namely, class Deltaproteobacteria (odds ratio [OR] = 0.762, 95% confidence interval [CI]: 0.612–0.949, *p* = 0.016), order Desulfovibrionales (OR = 0.755, 95% CI: 0.602–0.949, *p* = 0.016), family Desulfovibrionaceae (OR = 0.697, 95% CI: 0.544–0.892, *p* = 0.004), and genus *Coprococcus 2* (OR = 0.697, 95% CI: 0.552–0.879, *p* = 0.002), and the other two exhibited a promoting effect on HZ, i.e., phylum Cyanobacteria (OR = 1.59, 95% CI: 1.228–1.464, *p* = 0.022) and genus *Eubacterium rectale* (OR = 1.228, 95% CI: 1.031–1.464, *p* = 0.021) (Figure 2; Supplementary Table S1). Details of the instrumental variables are shown in Supplementary Table S2.

### 3.2 Causal effects of the gut microbiota on PHN

**FIGURE 3:**
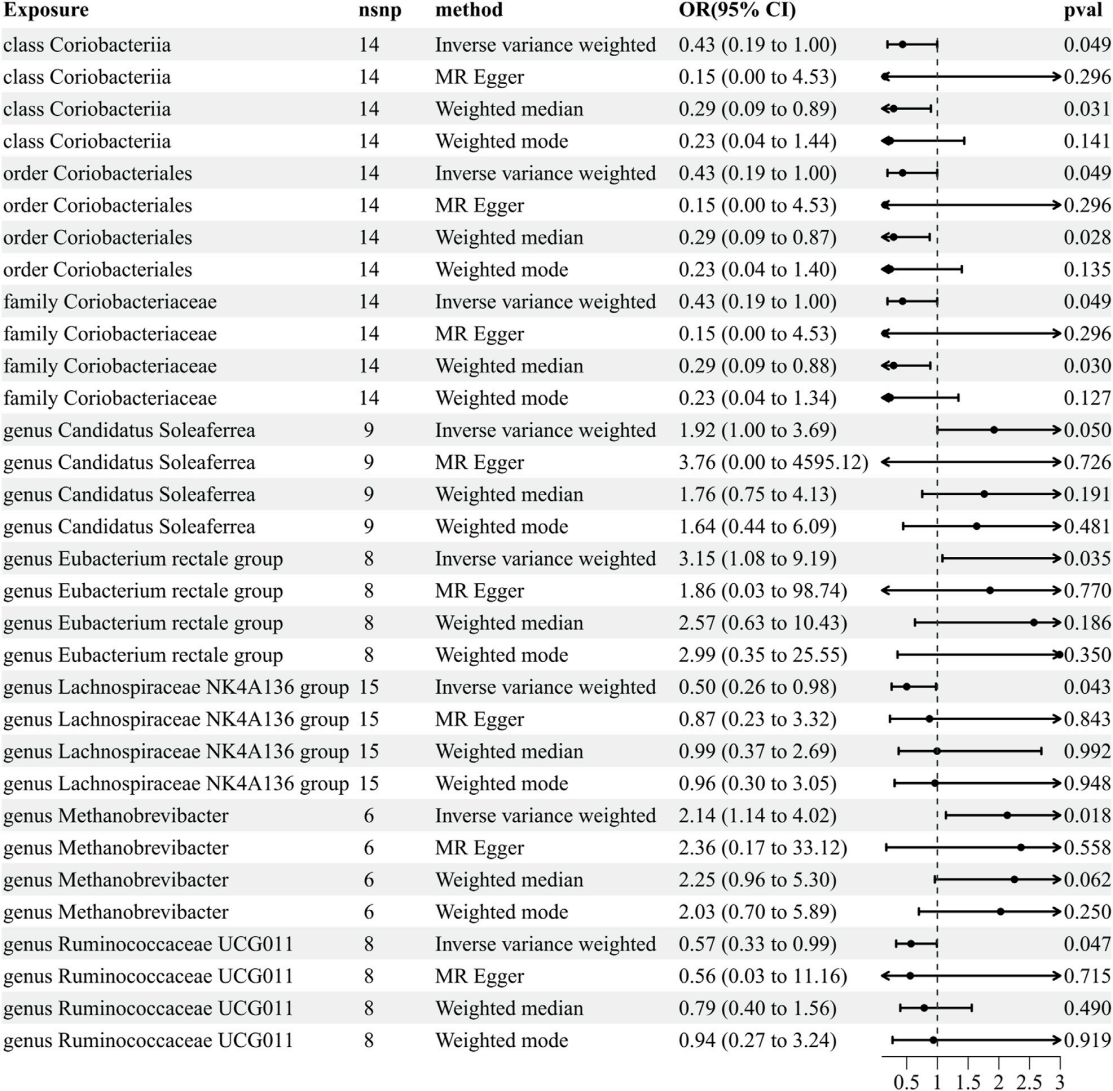
Results of MR analysis estimating the causal association between the gut microbiota and postherpetic neuralgia. The forward MR uses the gut microbiota as the exposure variable and postherpetic neuralgia risk as the outcome variable, and using the IVW method as the main result, effect sizes with 95% confidence intervals, the number of SNPs, and *p*-values were presented. CI, confidence interval; IVW, inverse-variance weighted; MR, Mendelian randomization; OR, odds ratio; PHN, postherpetic neuralgia.

Our comprehensive MR analysis revealed that eight bacterial traits exhibited statistically significant associations with PHN. These IVs were found in a diverse set of taxa, including 1 class (with 14 SNPs), 1 order (with 14 SNPs), 1 family (with 14 SNPs), and 5 genera (with 46 SNPs). Notably, each SNP demonstrated adequate validity, with all *F*-values were greater than 10. The IVW results showed that five bacterial genera were protective against PHN, namely, class Coriobacteriia (OR = 0.433, 95% CI: 0.187–0.998, *p* = 0.049), order Coriobacteriales (OR = 0.433, 95% CI: 0.187–0.998, *p* = 0.049), family Coriobacteriaceae (OR = 0.433, 95% CI: 0.187–0.998, *p* = 0.049), genus *Lachnospiraceae NK4A136* (OR = 0.500, 95% CI: 0.255–0.978, *p* = 0.043), and genus *Ruminococcaceae UCG011* (OR = 0.570, 95% CI: 0.327–0.992, *p* = 0.047), and the other three exhibited a promoting effect on PHN, i.e., genus *Candidatus Soleaferrea* (OR = 1.921, 95% CI: 1.082–9.191, *p* = 0.049), genus *Eubacterium rectale* group (OR = 1.51, 95% CI: 1.02–2.25, *p* = 0.035), and genus Methanobrevibacter (OR = 2.137, 95% CI: 1.137–4.016, *p* = 0.018) (Figure 3; Supplementary Table S3). Details of the instrumental variables are shown in Supplementary Table S2.

### 3.3 Reverse MR analyses

**FIGURE 4:**
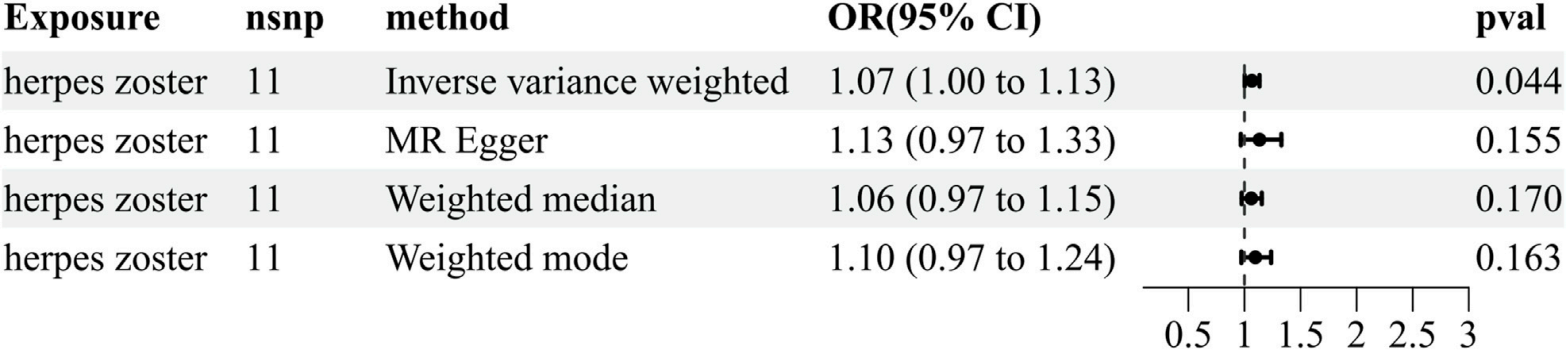
Reverse results of MR analysis estimating the causal association between herpes zoster and the gut microbiota. CI, confidence interval; IVW, inverse-variance weighted; MR, Mendelian randomization; OR, odds ratio.

To further exclude reverse causality, we extracted IVs from HZ (the six identified microorganisms) and PHN (the eight identified microorganisms) as an exposure to explore the influence of HZ and PHN on the gut microbiota (Supplementary Table S2). We found that HZ has a positive effect on the genus *Eubacterium rectale* group (OR = 1.066, 95% CI: 1.002–1.134, *p* = 0.044) (Figure 4; Supplementary Table S4). The reverse MR analyses failed to provide evidence supporting the causal effects of genetically proxied PHN on the eight identified microorganisms (Supplementary Table S5). Details of the instrumental variables are shown in Supplementary Table S6.

### 3.4 Reliability of data

Cochran’s Q test indicated that there was no heterogeneity in the IVs in both MR–Egger and IVW analysis results (*p* > 0.05). In the MR–Egger intercept analysis, no significant pleiotropy was observed (*p* > 0.05). Meanwhile, the MR-PRESSO analysis did not identify any significant outliers, and this test also revealed no evidence of horizontal pleiotropy (*p* > 0.05). In addition, we conducted a “leave-one-out” analysis to identify possible heterogeneous SNPs. No SNP was found to significantly impact the results. To visualize data, we used the following graphical representations: 1) funnel plots (mostly used to detect heterogeneity among SNPs); 2) forest plots (show the effect of each SNP and its 95% confidence interval); and 3) scatter plots (each point in the scatter plot corresponds to an SNP, showing the association between an SNP and exposure or outcome). Details of the instrumental variables are shown in Supplementary Figures S1-S15.

## 4 Discussion

To the best of our knowledge, this study is the first to leverage a bidirectional, two-sample MR method to detect the relationship between the gut microbiota and HZ and PHN and the underlying mechanisms. The results obtained from our MR study present robust genetic evidence suggesting that changes in the composition of certain gut microbiota significantly contribute to the onset and progression of HZ and PHN. We found that the phylum Cyanobacteria, genus *Eubacterium rectale* group was a risk effect against HZ and genus *Candidatus Soleaferrea*, genus *Eubacterium rectale* group, and genus *Methanobrevibacter* were a risk effect against PHN, while class Deltaproteobacteria, order Desulfovibrionales, family Desulfovibrionaceae, and genus *Coprococcus 2* were negatively associated with HZ, suggesting a protective effect, and class Coriobacteriia, order Coriobacteriales, family Coriobacteriaceae, genus *Lachnospiraceae NK4A136*, and genus *Ruminococcaceae UCG011* were negatively associated with PHN. In addition, the onset of HZ changed the composition of the gut microbiota, and there was an increase in the level of the genus *Eubacterium rectale* group.

Based on preceding studies, *Coprococcus* was found to play a crucial role in aberrant immune responses and exhibited a considerable correlation with the severity of skin diseases such as urticaria (Xu et al., 2022; Shi et al., 2023). Inconsistent with the above research, this study found that genus *Coprococcus 2* was protective against HZ. Although both HZ and urticaria are inflammatory responses to the skin, there was a conflicting finding about the role played by genus *Coprococcus* in the two diseases. We deduce that the genus *Coprococcus* can modulate skin inflammation through diverse immune pathways. Meanwhile, our study also showed that class Deltaproteobacteria, order Desulfovibrionales, and family Desulfovibrionaceae, which all belong to phylum Proteobacteria, are negatively correlated with HZ. Desulfovibrionales and Desulfovibrionaceae, which can reduce sulfate to H_2_S, cause damage to the intestinal barrier, resulting in the production of harmful substances like endotoxins and pro-inflammatory cytokines (Wei et al., 2023). Based on pre-clinical and clinical trial data, it has been observed that Proteobacteria interact with some dietary components, leading to the production of pro-inflammatory cytokines. Ultimately, this interaction further contributes to the occurrence of gastrointestinal inflammation (Faqerah et al., 2023). A previous study revealed that Deltaproteobacteria were negatively related to anti-neutrophil cytoplasmic antibody-associated vasculitis with kidney injury (Yu et al., 2022). The inconsistent outcomes observed among diverse microbiota act as a reminder that there is a notable inter- and intra-species diversity, which exerts a significant influence on the overall health of the host.

Class Coriobacteriia and its child taxa, order Coriobacteriales, and family Coriobacteriaceae all are negatively associated with PHN. However, there are no studies on the relationship between these bacterial taxa and PHN and on whether these bacteria have a concert effect, and the possible mechanisms remain to be explored. *Ruminococcaceae* is a dominant bacterial genus within the human gut, which is known to generate short-chain fatty acids (SCFAs). SCFAs enhance the integrity of the intestinal epithelial barrier and exert anti-inflammatory effects on epithelial cells (La Reau and Suen, 2018; Chen et al., 2023). Our findings provide evidence for the protective effect of *Ruminococcaceae* against PHN, which may be attributed to the role of SCFAs in the inhibition of inflammatory response. As for genus *Lachnospiraceae*, evidence from various research studies shows that it might influence healthy functions and increase in diseases such as metabolic syndrome, obesity, diabetes, liver diseases, and IBD (Sun et al., 2021; Awoniyi et al., 2023; Zang et al., 2023). Conversely, our study showed that genus *Lachnospiraceae* can reduce the risk of PHN, which highlights the fact that different diseases have varied mechanisms of pathogenesis.

In this research, we found that phylum Cyanobacteria exhibited a promoting effect on HZ. Cyanobacteria are a part of the intestinal microbiota, the abundance of which tends to increase in patients with selected neurodegenerative diseases (Hu and Rzymski, 2022). Currently, there are few direct studies on Cyanobacteria and HZ or PHN. However, studies have found that PHN and neurodegenerative diseases share common mechanisms and pathological processes, such as abnormal activity of voltage-gated sodium channels (Zuliani et al., 2010) and the replication of HZ virus within brain cells, contributing to the development of neurodegenerative diseases (Choi et al., 2021). Therefore, we speculate that an increase in phylum Cyanobacteria abundance is also one of the risk factors for HZ similar to neurodegenerative diseases. Genus *Candidatus Soleaferrea* exerts anti-inflammatory effects by secreting metabolites and maintaining a stable intestinal environment (Cai et al., 2020). However, our study results showed that *Candidatus Soleaferrea* significantly increased the risk of PHN. Moreover, *Methanobrevibacter*, which are beneficial butyrate producers, show a significant decrease in their abundance within the intestinal tract of inflammatory bowel disease patients (Danilova et al., 2019). We found that *Methanobrevibacter* have a promoting effect on PHN in this study. These results appear to contradict those of previous studies. Possible reasons for this may include differences in the racial composition of the study population, small sample sizes, and the diversity of research methodologies. Future studies with more precision are needed to uncover the association between this microbiota and PHN.

Interestingly, it should be noted that the genus *Eubacterium rectale* group in our study showed a promoting effect on both HZ and PHN. *Eubacterium rectale*, as an important member of the human gut microbiota, has been studied in many research studies. A study showed that the composition of the gut microbiota in Chinese Prader–Willi syndrome patients characterized by increased *Eubacterium rectale* is different from that in patients with obesity (Zhong et al., 2023b). Animal experiments of mice showed that the *Eubacterium rectale* group can promote systemic inflammatory responses by inhibiting the expression of CD83 (Islam et al., 2021). Wang et al. reported that in normal colonic epithelial cells, the endotoxin of the *Eubacterium rectale* group activates the transcription factor NF-κΒ, participating in the regulation of processes such as innate and adaptive immune responses, and also plays a pro-inflammatory role in colorectal cancer (Wang et al., 2021). Therefore, we could infer that the *Eubacterium rectale* group may have a promoting effect on HZ and PHN through systemic inflammation. Meanwhile, in our study, we also found that HZ has a positive effect on the genus *Eubacterium rectale* group, which demonstrates the existence of a bidirectional causal relationship between the gut microbiota and HZ. These results may provide novel evidence supporting that this microbiota can be a supplementary biomarker of the diagnosis or treatment of HZ and PHN.

Based on our study results, we aim to incorporate these findings into clinical practice, public health, or future areas of research. First, our findings underscore the role of the gut microbiota in the onset of HZ and PHN, which may prompt clinicians to pay more attention to a patient’s gut health during the treatment. For instance, clinicians might consider the use of probiotics or prebiotics to modulate the patient’s gut microbiota, aiming to ameliorate the condition or alleviate pain. Second, on a public health level, our findings emphasize the importance of maintaining a balanced gut microbiota, which could aid in the prevention of HZ and PHN. For example, public health policymakers may promote healthy lifestyles, including a balanced diet and moderate exercise, to maintain gut microbiota health. Furthermore, future investigations could assess whether the regulation of gut microbiota balance (e.g., through the use of probiotics or prebiotics) is effective in preventing the occurrence of HZ and PHN or improving the condition.

Although our study suggests possible correlations between the gut microbiota and HZ and PHN, additional functional investigations may clarify the ways in which the identified gut microbiota impact the development of HZ and PHN. When interpreting the results of our study, it is important to recognize and address several limitations. First, in order to enhance the sample size, the original dataset of the gut microbiota incorporated data on 24 distinct ethnic cohorts, and the summary statistics of HZ and PHN were from a European population, which may introduce a potential bias in the genetic association and population stratification. To address this concern, it is recommended to undertake future GWASs with larger sample sizes, a more homogeneous ethnic population, and standardized sequencing techniques. Second, a number of sensitivity analyses were carried out to assess the robustness of the results. We chose a *p*-value < 1 × 10^−5^ as a looser threshold to bring in more SNPs as IVs, and the selection may account for a minuscule portion of the variance in exposure. Third, insufficient information was available regarding the severity of HZ and PHN, as well as symptoms like allodynia and nociceptive hypersensitivity, and subgroup analysis could not be conducted in this study. Meanwhile, it should be remembered that the pathway from the exposure to outcome is extremely complex, the occurrence of HZ and PHN is determined by many factors, and the biological function of many genetic instrumental variables is still unknown.

In conclusion, using publicly accessible gene databases, our study confirmed the causal relationship between gut microbiota and HZ and PHN. It is worth noting that the genus *Eubacterium rectale* group displayed a significant risk effect against HZ and PHN, and the reverse MR analysis revealed that HZ had a positively causal effect on it. This makes the genus *Eubacterium rectale* group a potential new target for the diagnosis and treatment of HZ. To validate the causal linkages over time, future research could concentrate on conducting longitudinal studies or reproducing these findings in other ethnically varied populations.

## Data Availability

The original contributions presented in the study are included in the article/Supplementary Material; further inquiries can be directed to the corresponding authors.
