# Supplementary figures and images for: Genetic insights into the gut microbiota, herpes zoster, and postherpetic neuralgia: a bidirectional two-sample Mendelian randomization study
Source: Front Genet. 2024 May 23;15:1366824. doi: 10.3389/fgene.2024.1366824 (PMC11153692; doi:10.3389/fgene.2024.1366824)

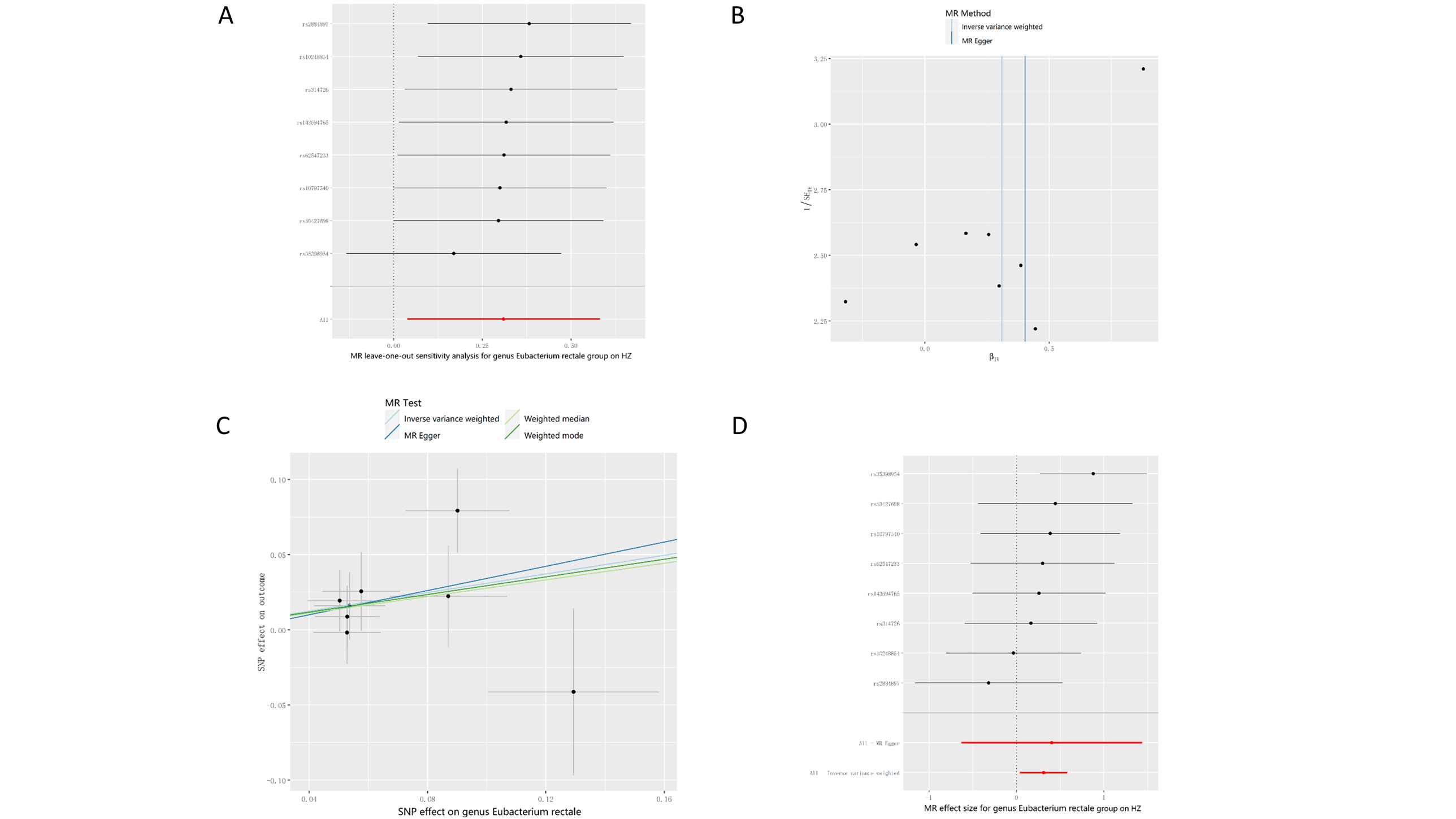

Supplement: Supplementary file 1 [file Image6.TIF]

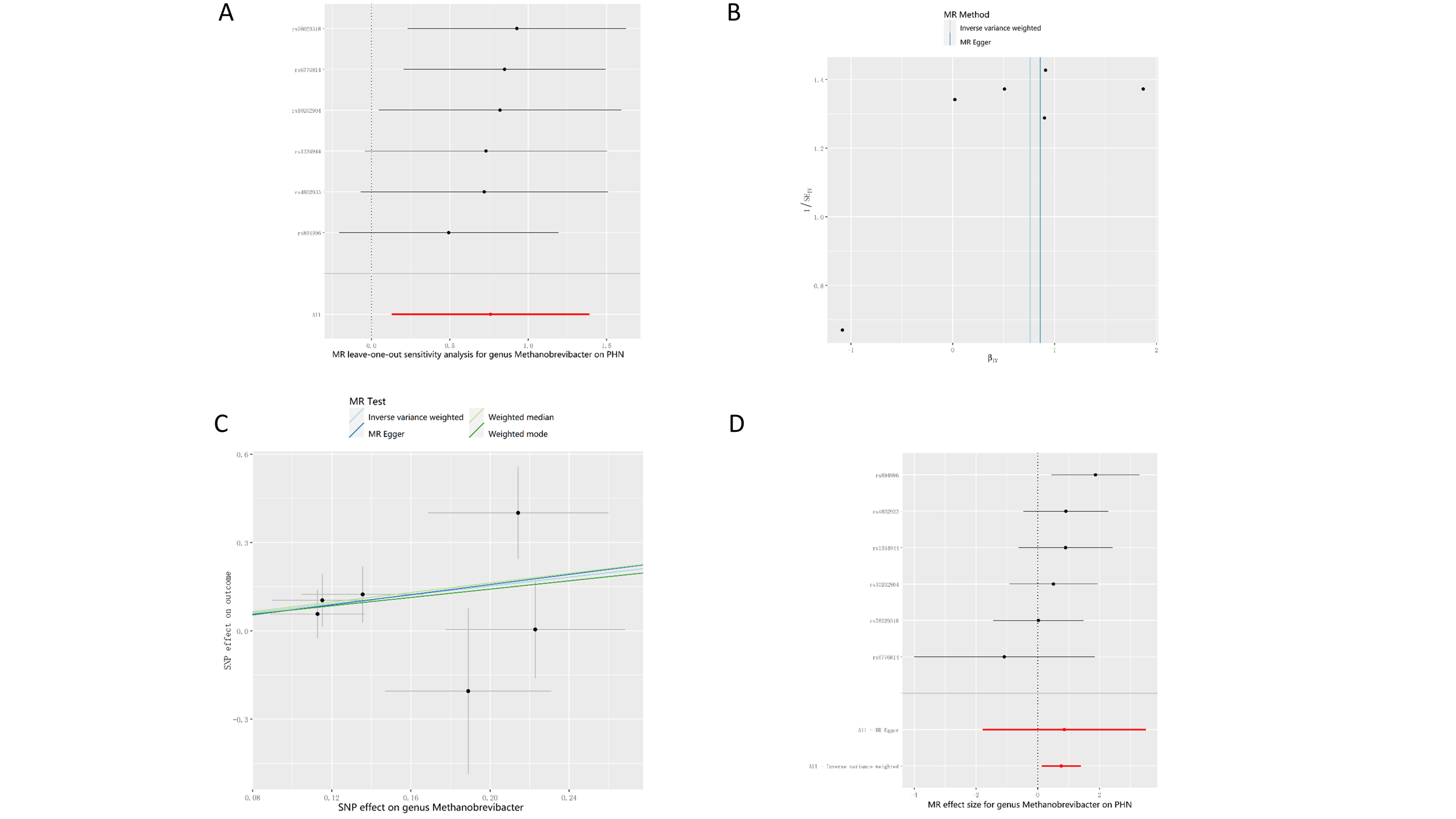

Supplement: Supplementary file 2 [file Image14.TIF]

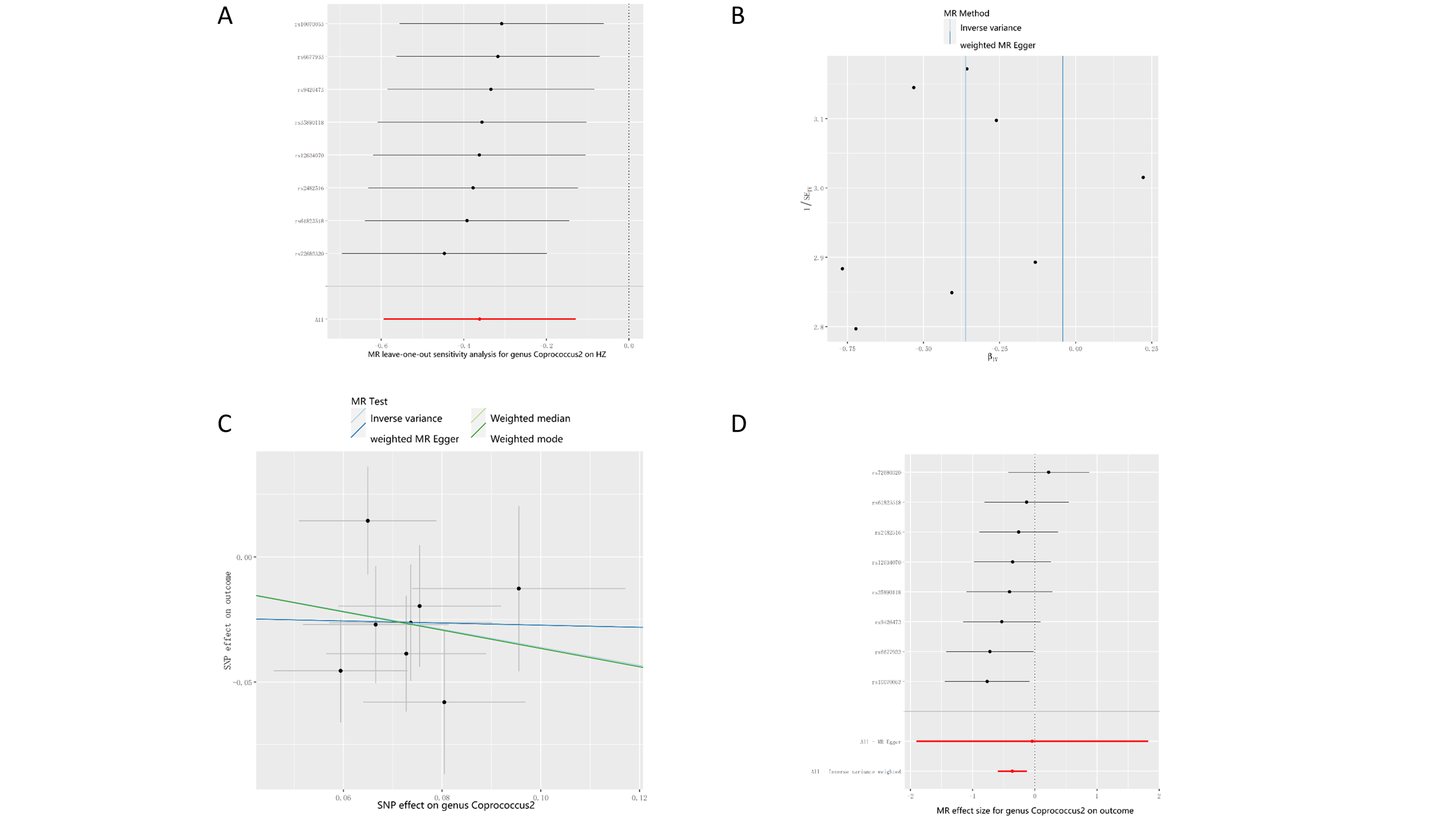

Supplement: Supplementary file 3 [file Image3.TIF]

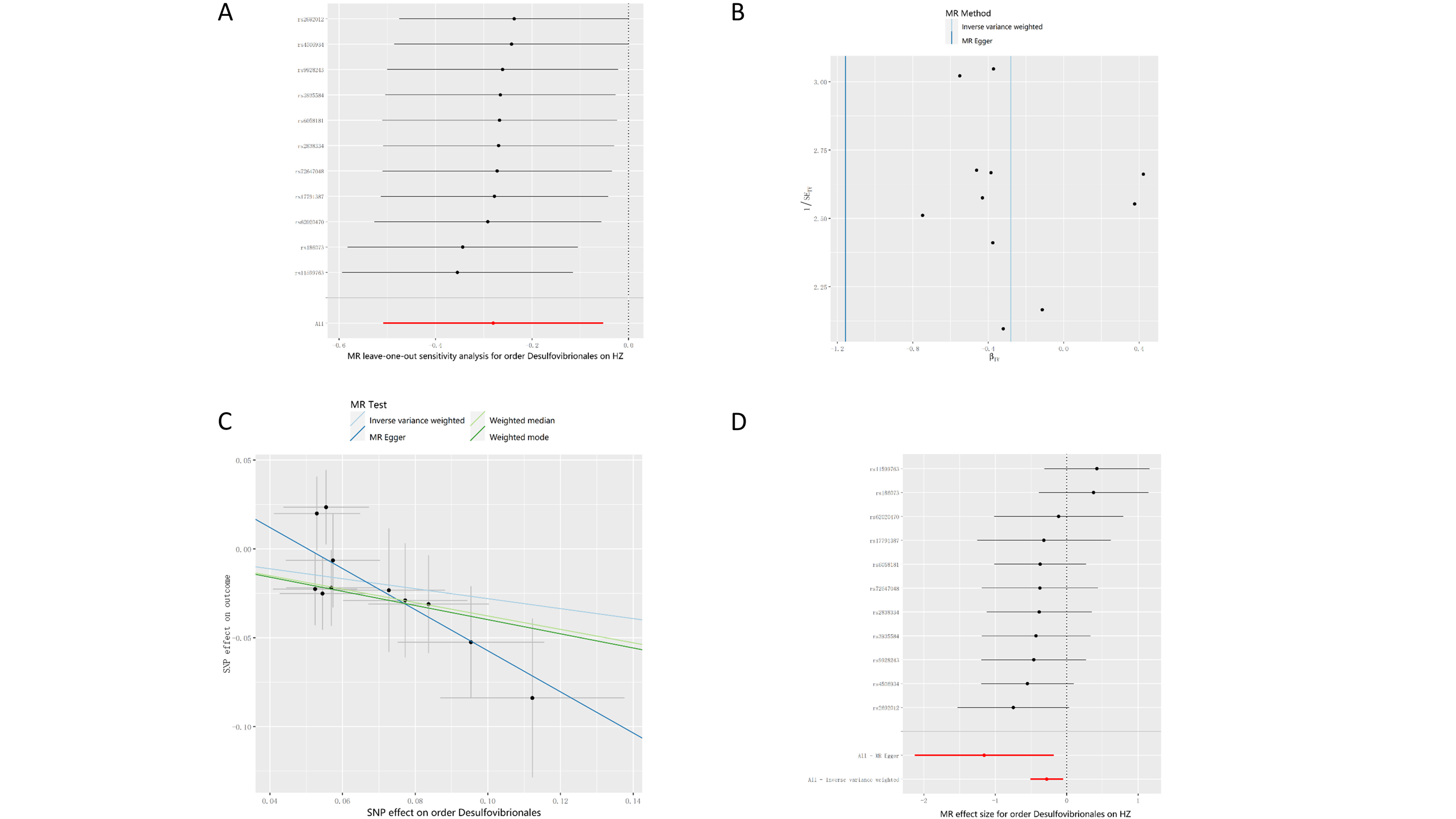

Supplement: Supplementary file 4 [file Image4.TIF]

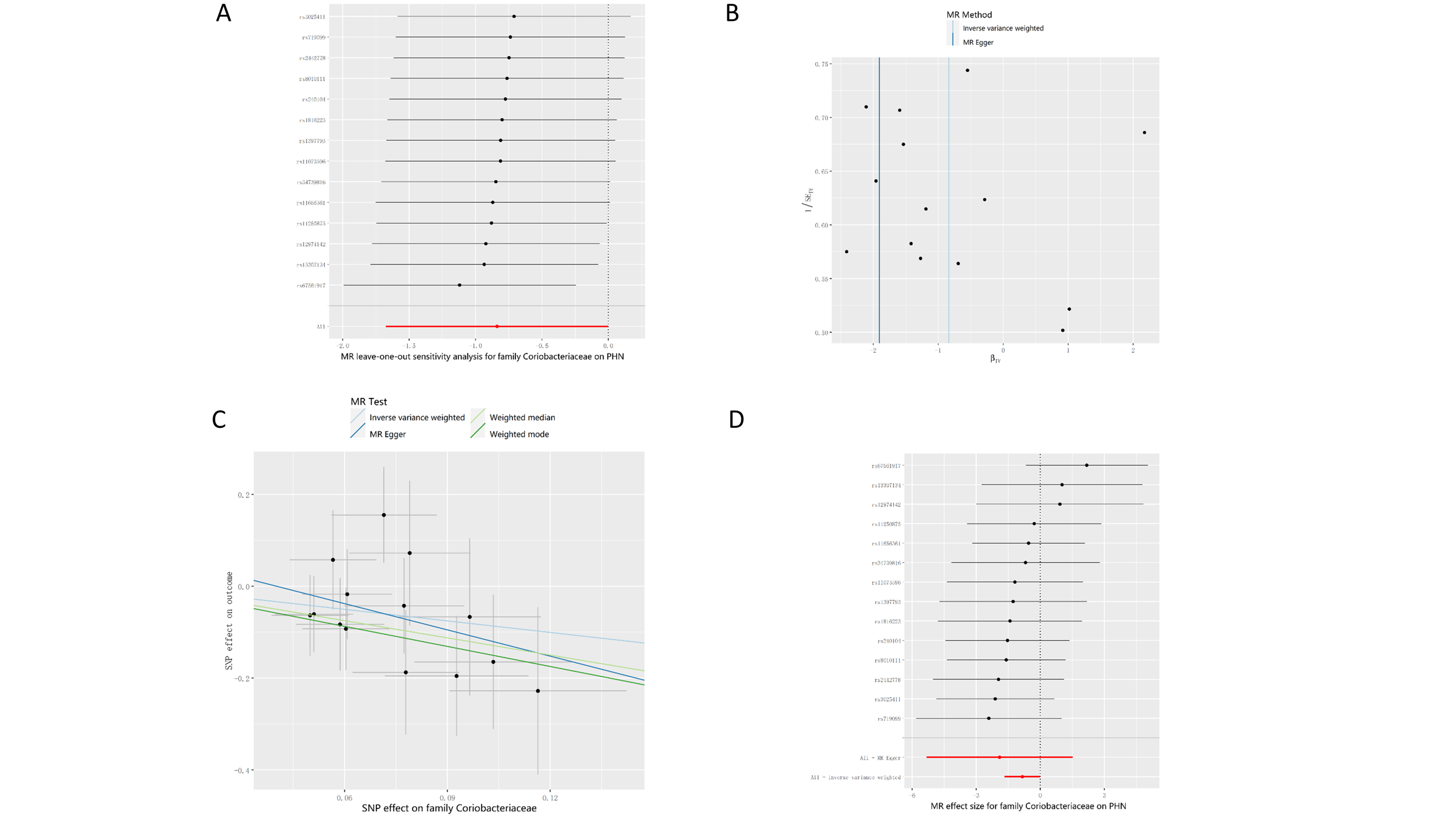

Supplement: Supplementary file 5 [file Image9.TIF]

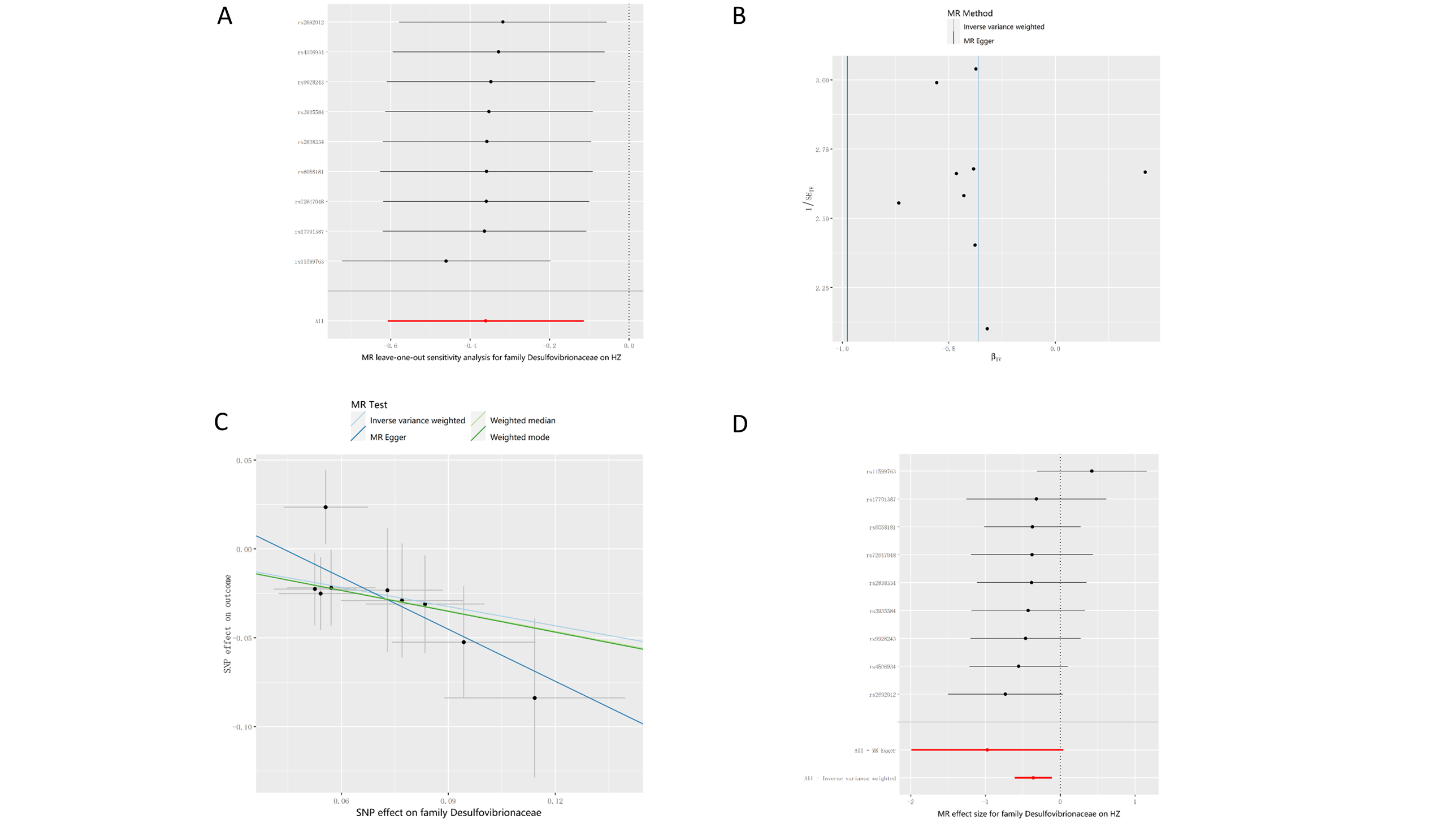

Supplement: Supplementary file 6 [file Image2.TIF]

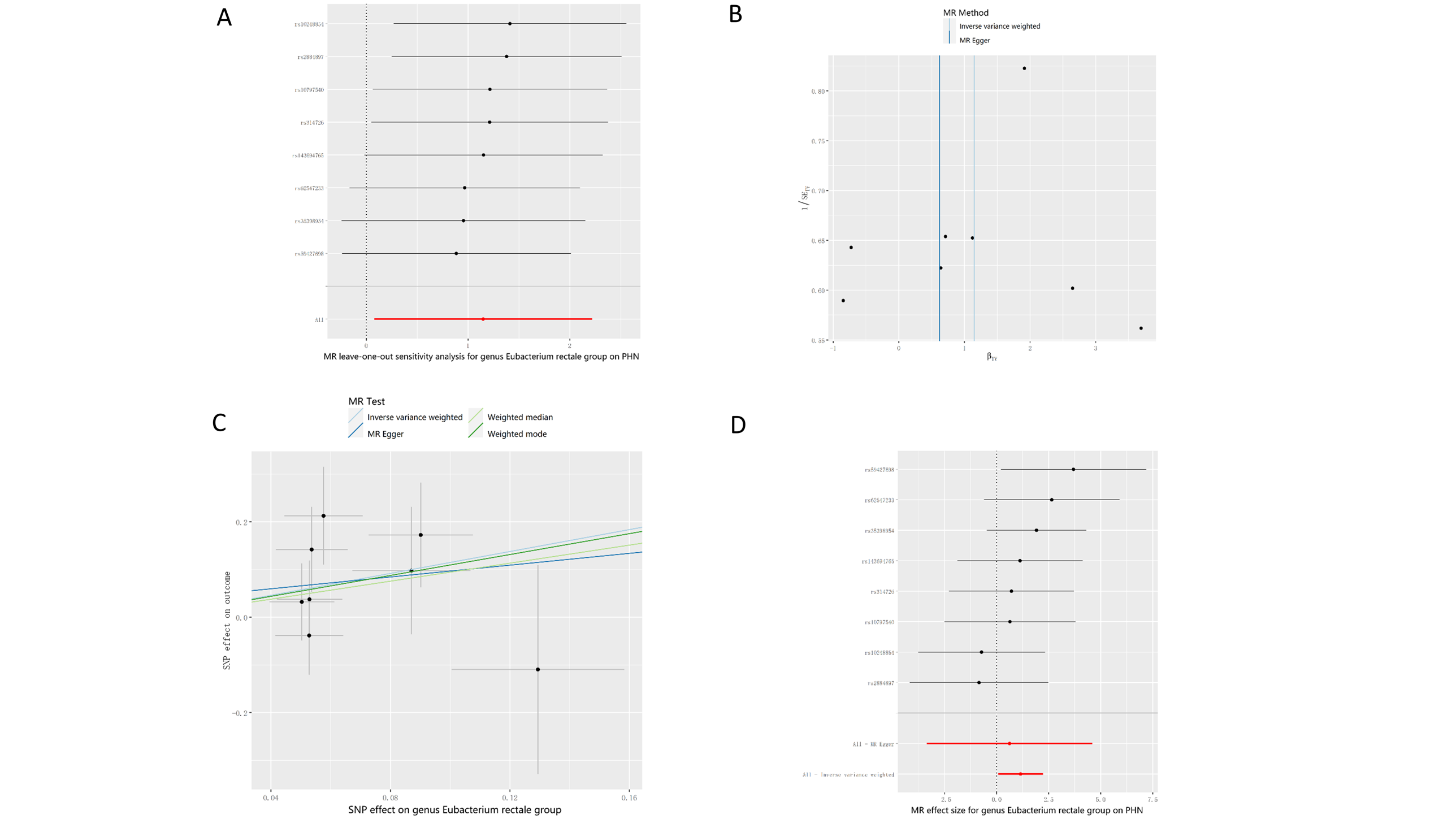

Supplement: Supplementary file 7 [file Image13.TIF]

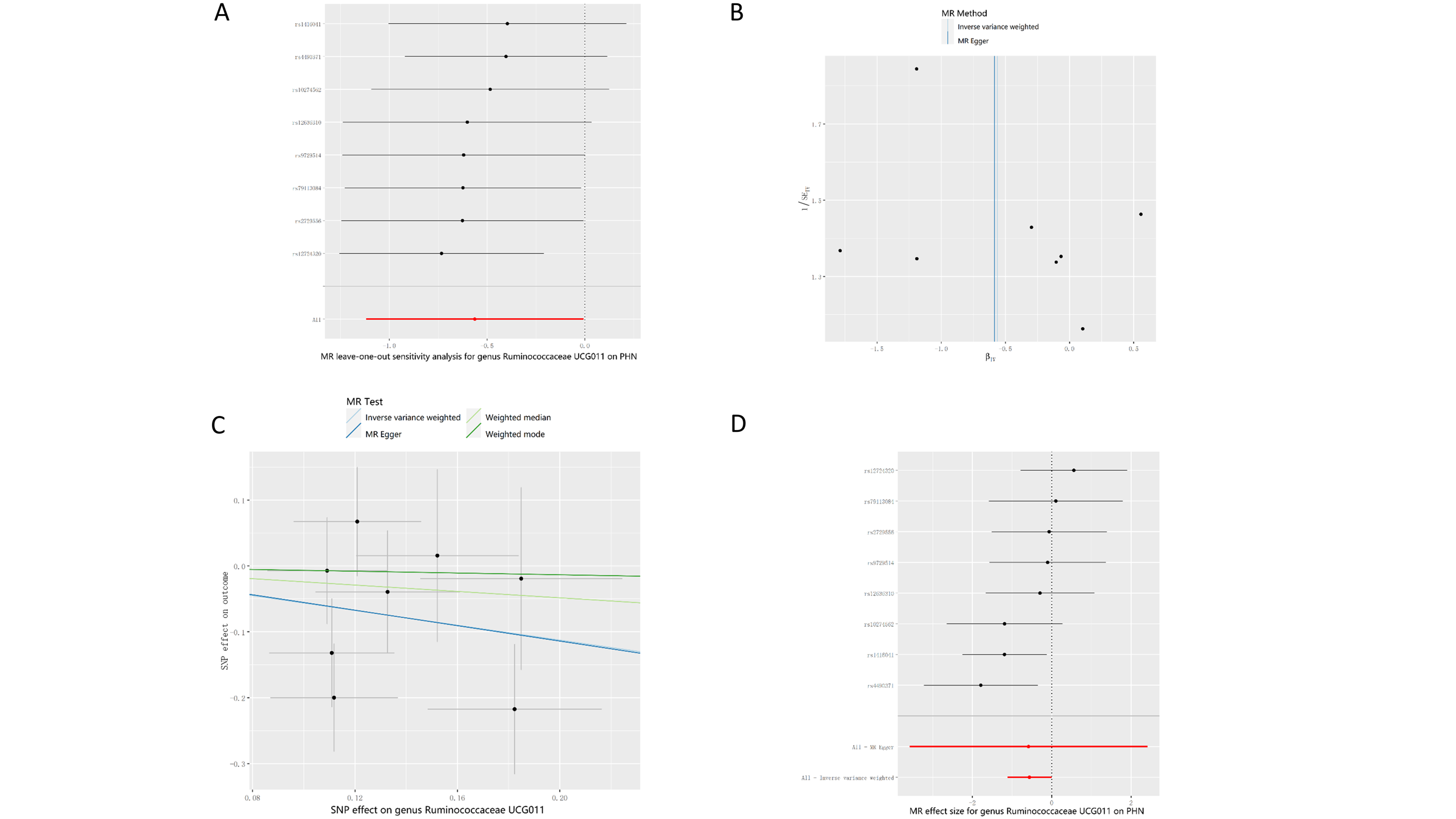

Supplement: Supplementary file 8 [file Image11.TIF]

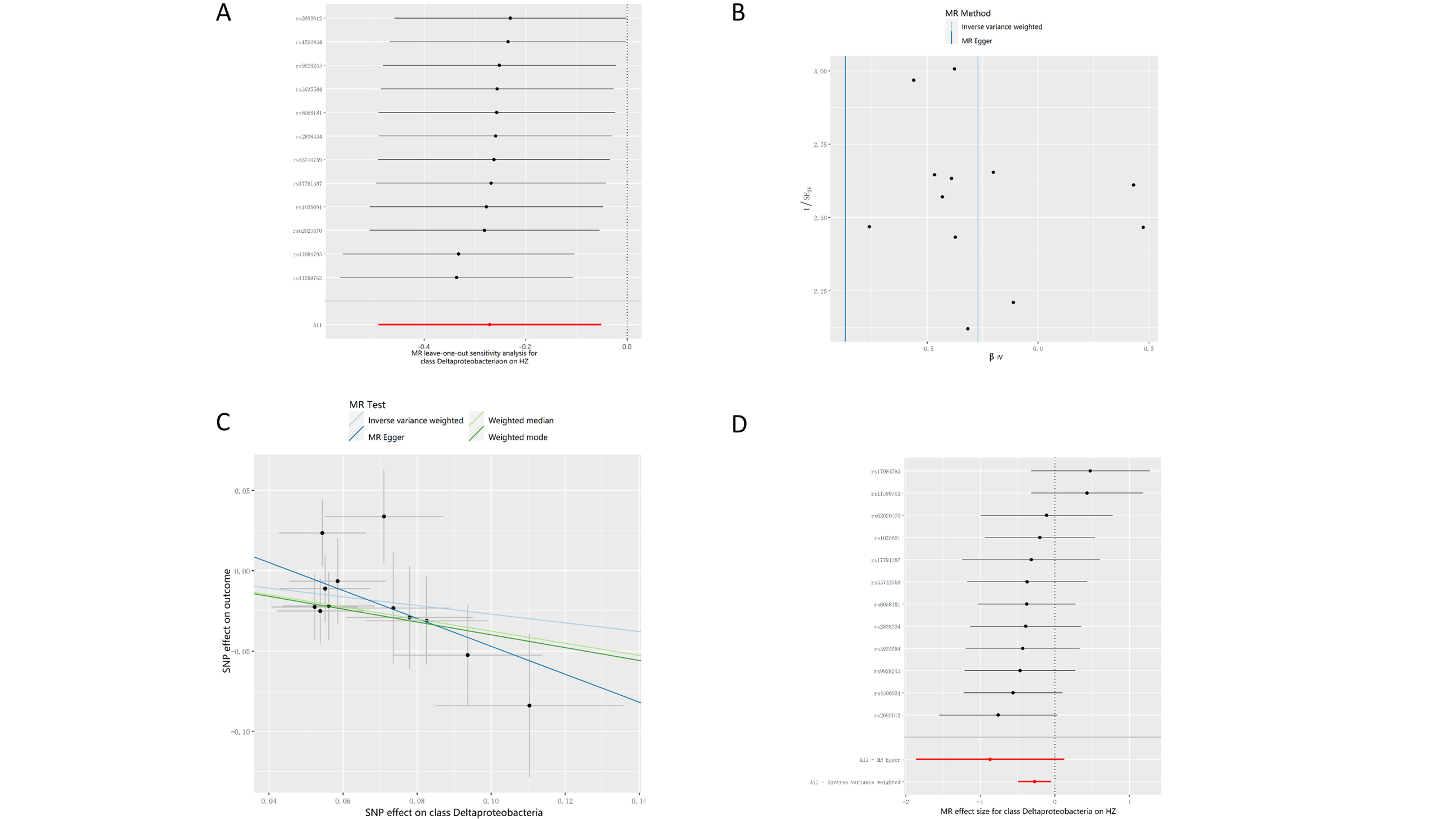

Supplement: Supplementary file 9 [file Image1.TIF]

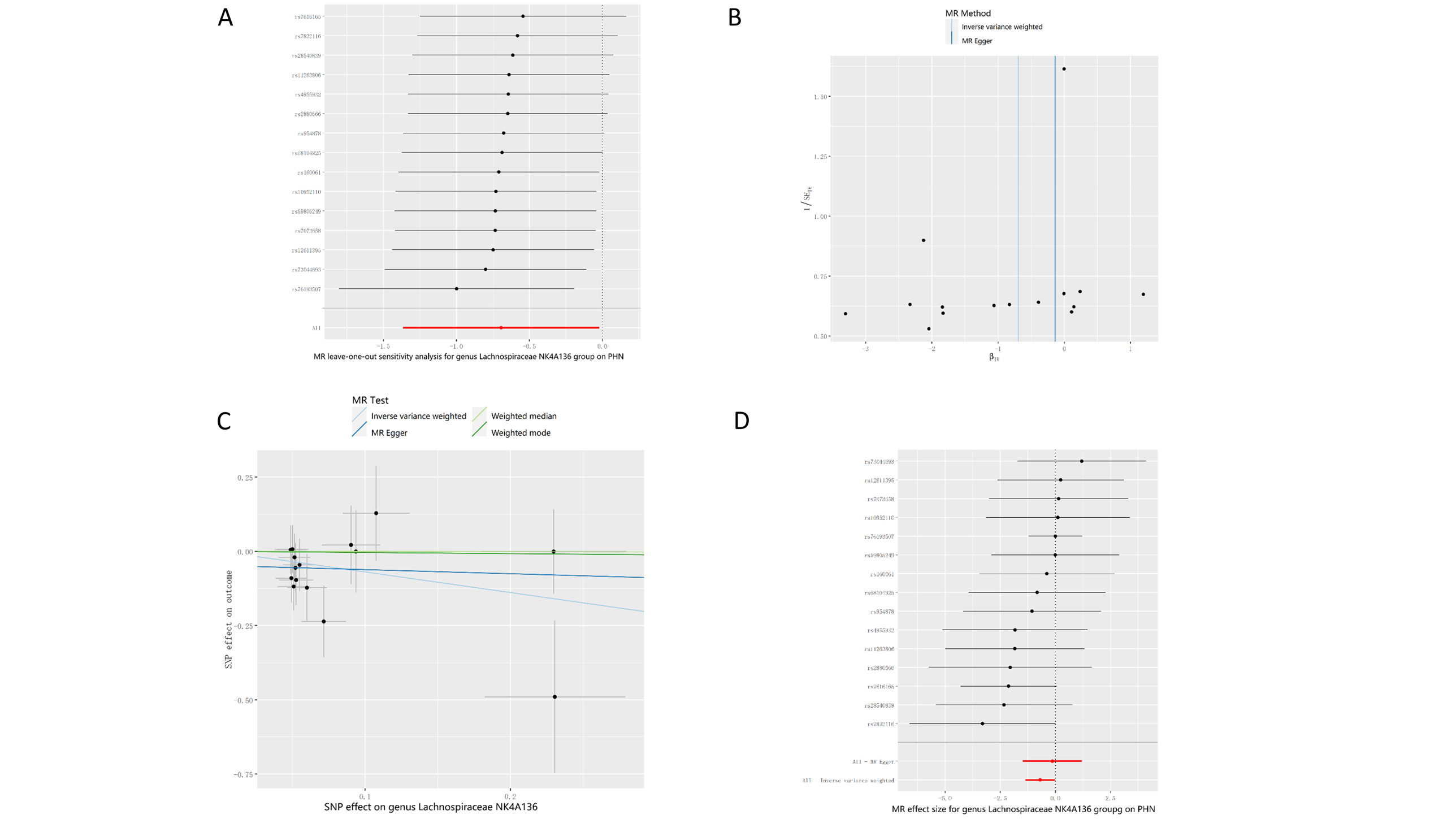

Supplement: Supplementary file 10 [file Image10.TIF]

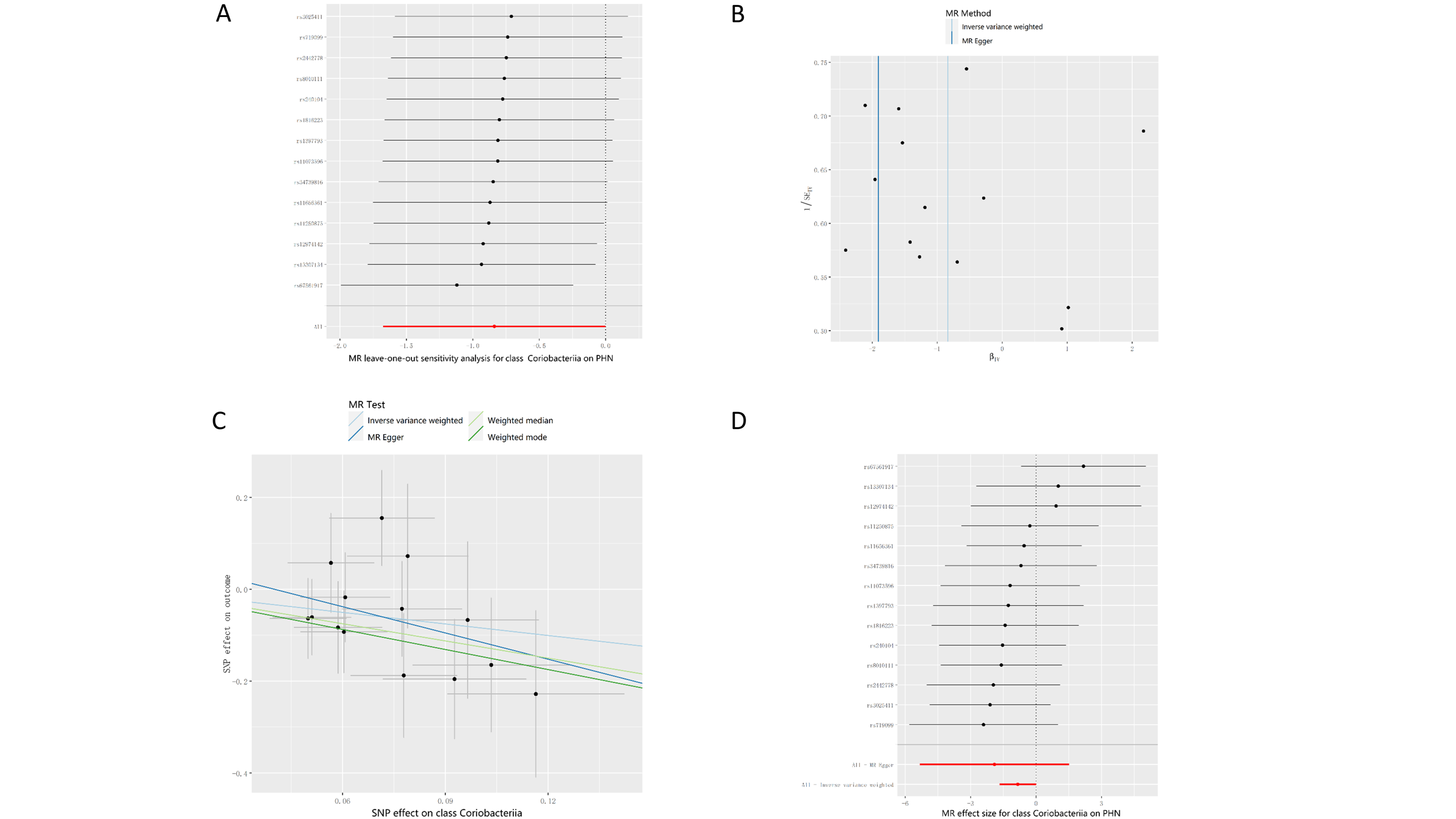

Supplement: Supplementary file 11 [file Image7.TIF]

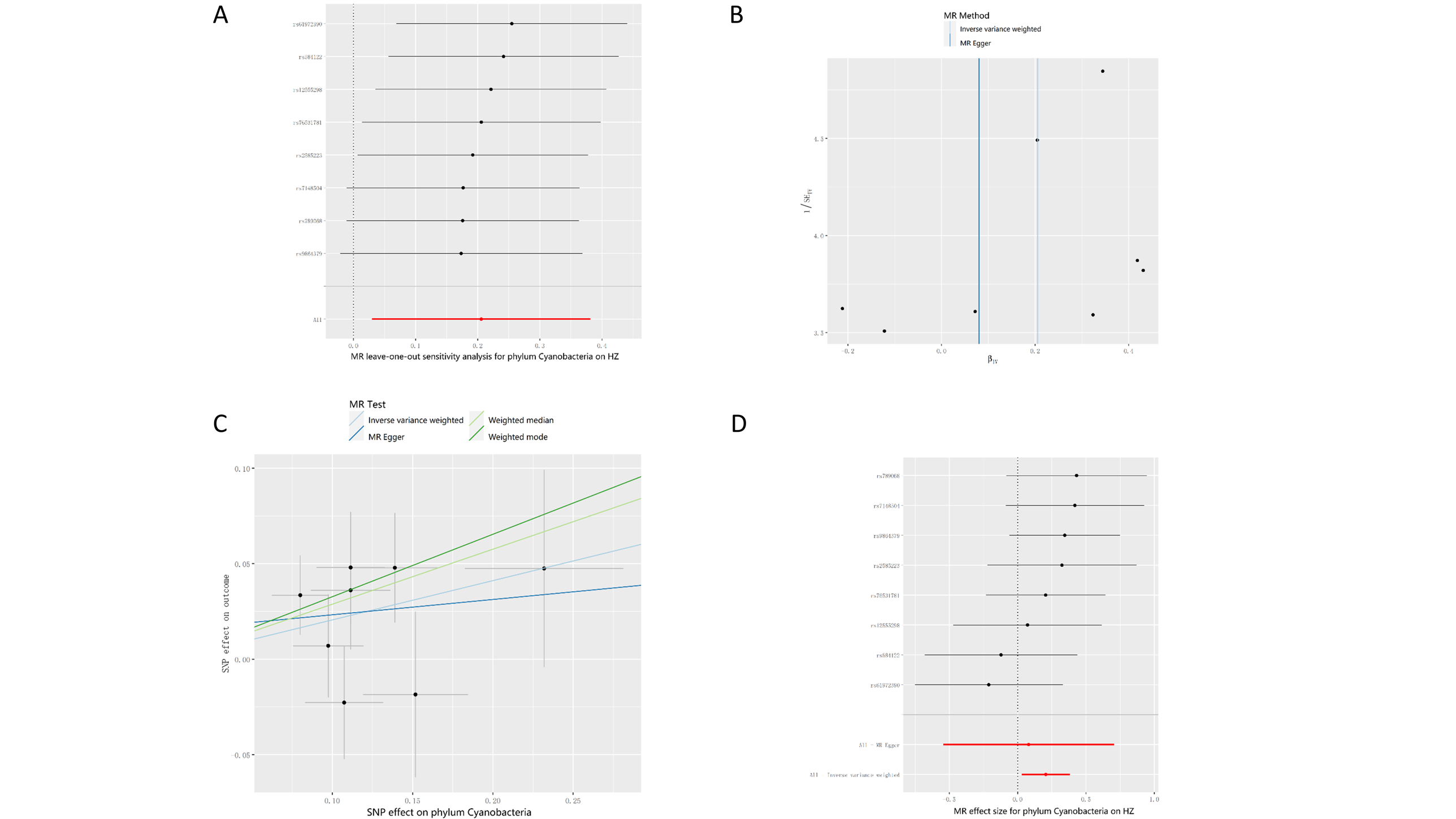

Supplement: Supplementary file 15 [file Image5.TIF]

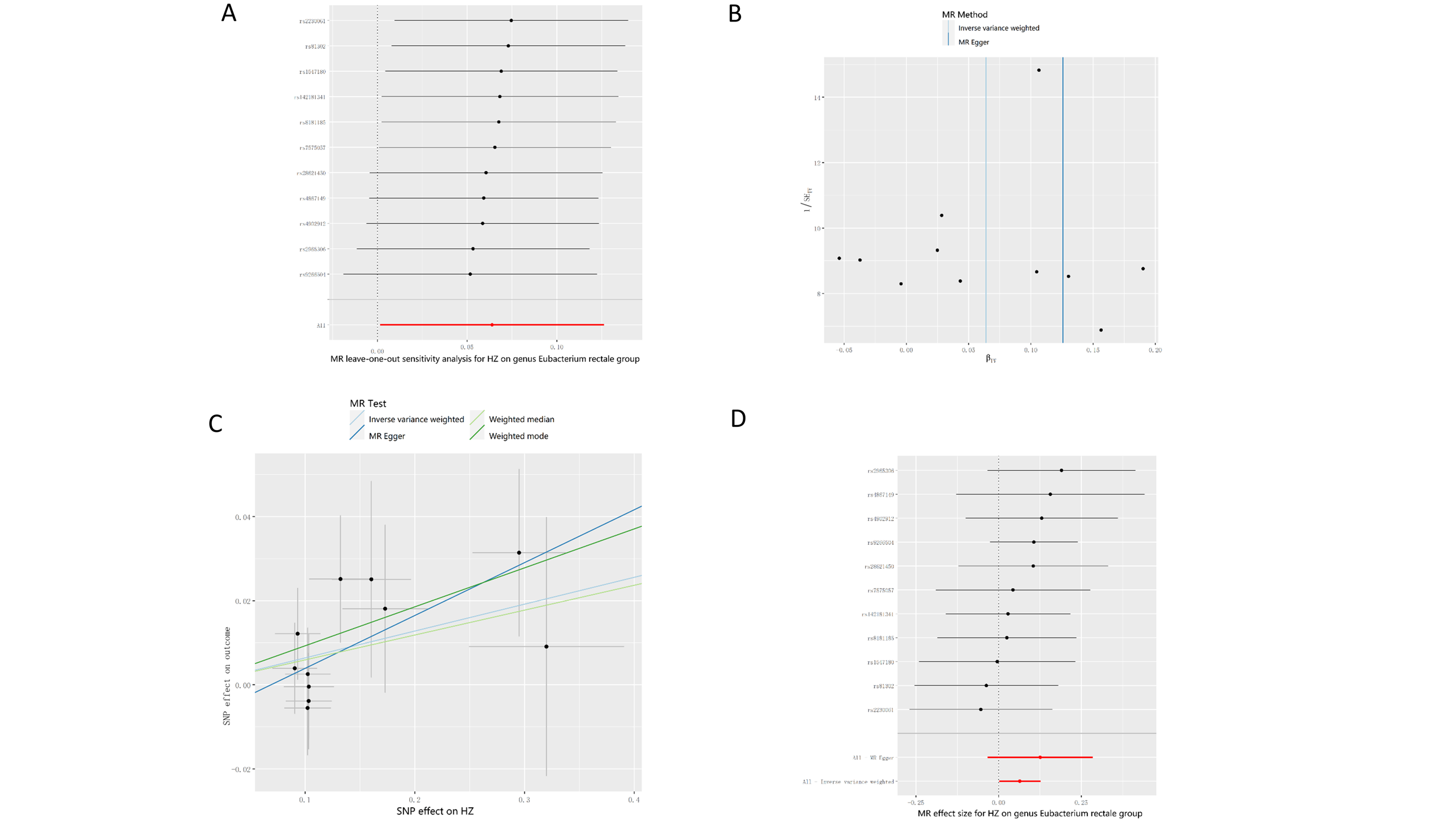

Supplement: Supplementary file 16 [file Image15.TIF]

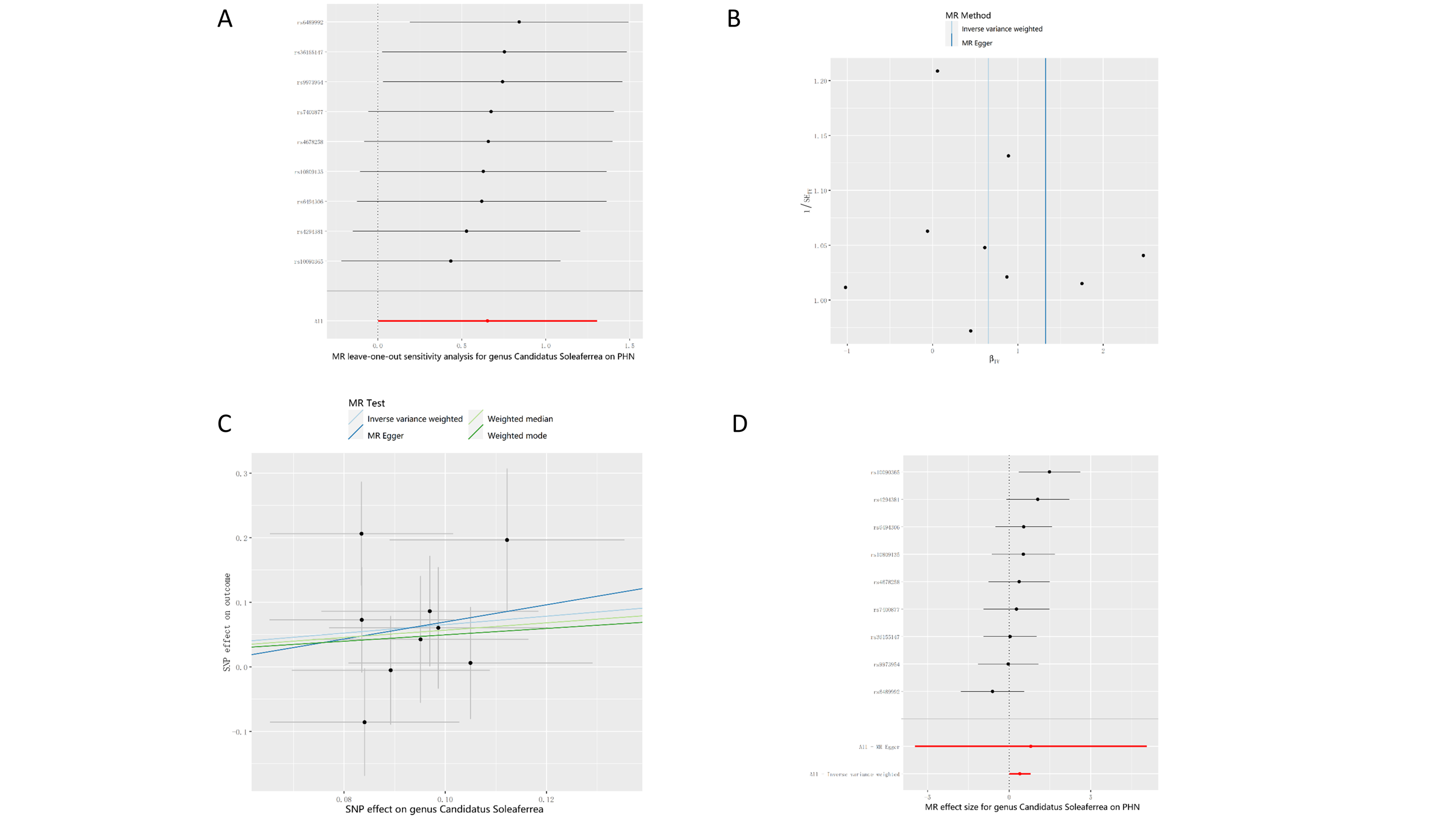

Supplement: Supplementary file 17 [file Image12.TIF]
