# Supplementary material for: Genetic insights into the gut microbiota, herpes zoster, and postherpetic neuralgia: a bidirectional two-sample Mendelian randomization study
Source: Front Genet. 2024 May 23;15:1366824. doi: 10.3389/fgene.2024.1366824 (PMC11153692; doi:10.3389/fgene.2024.1366824)
Supplement: Supplementary file 12 [file Table2.DOCX]

**Supplementary Table 1** MR estimates for the association between gut microbiota and HZ

| Exposure | Outcome | NO.of  SNP | Methods | *β* | | *OR* | | *P* | | *P* of  Pleiotropy | | *P* of  Heterogeneity | |
| --- | --- | --- | --- | --- | --- | --- | --- | --- | --- | --- | --- | --- | --- |
| class Deltaproteobacteria | HZ | 12 | IVW | | -0.271 | | 0.762 | | 0.016 | | 0.255 | | 0.595 |
|  |  | 12 | MR-Egger | | -0.869 | | 0.419 | | 0.510 | |  | |  |
|  |  | 12 | Weighted median | | -0.377 | | 0.686 | | 0.421 | |  | |  |
|  |  | 12 | Weighted mode | | -0.399 | | 0.671 | | 0.602 | |  | |  |
| order Desulfovibrionales |  | 11 | IVW | | -0.280 | | 0.755 | | 0.016 | | 0.103 | | 0.760 |
|  |  | 11 | MR-Egger | | -1.157 | | 0.314 | | 0.045 | |  | |  |
|  |  | 11 | Weighted median | | -0.377 | | 0.685 | | 0.018 | |  | |  |
| family Desulfovibrionaceae  genus Coprococcus2  phylum Cyanobacteria  genus Eubacterium rectale group |  | 11  9  9  9  9  8  8  8  8  8  8  8  8  8  8  8  8 | Weighted mode  IVW  MR-Egger  Weighted median  Weighted mode  IVW  MR-Egger  Weighted median  Weighted mode  IVW  MR-Egger  Weighted median  Weighted mode  IVW  MR-Egger  Weighted median  Weighted mode | | -0.399  -0.361  -0.976  -0.387  -0.391  -0.362  -0.976  -0.387  -0.391  0.206  0.080  0.288  0.327  0.310  0.403  0.277  0.293 | | 0.671  0.697  0.377  0.679  0.676  0.697  0.958  0.693  0.693  1.228  1.083  1.333  1.387  1.363  1.496  1.319  1.340 | | 0.131  0.004  0.102  0.022  0.134  0.002  0.966  0.022  0.183  0.022  0.810  0.014  0.101  0.025  0.474  0.154  0.379 | | 0.261  0.747  0.696  0.860 | | 0.752  0.401  0.454  0.371 |

Abbreviations: HZ- herpes zoster; IVW-inverse variance weighting; MR-mendelian randomization; OR—odds ratio.

**Supplementary Table 3** MR estimates for the association between gut microbiota and PHN

| Exposure | Outcome | NO.of  SNP | Methods | *β* | | *OR* | | *P* | | *P* of  Pleiotropy | | *P* of  Heterogeneity | |
| --- | --- | --- | --- | --- | --- | --- | --- | --- | --- | --- | --- | --- | --- |
| class Coriobacteriia  order Coriobacteriales  family Coriobacteriaceae  genus Lachnospiraceae NK4A136 group  genus Ruminococcaceae UCG011  genus Candidatus Soleaferrea  genus Eubacterium rectale group  genus Methanobrevibacter | PHN | 14  14  14  14  14  14  14  14  14  14  14  14  15  15  15  15  8  8  8  8  9  9  9  9  8  8  8  8  6  6  6  6 | IVW  MR-Egger  Weighted median  Weighted mode  IVW  MR-Egger  Weighted median  Weighted mode  IVW  MR-Egger  Weighted median  Weighted mode  IVW  MR-Egger  Weighted median  Weighted mode  IVW  MR-Egger  Weighted median  Weighted mode  IVW  MR-Egger  Weighted median  Weighted mode  IVW  MR-Egger  Weighted median  Weighted mode  IVW  MR-Egger  Weighted median  Weighted mode | | -0.838  -1.906  -1.249  -1.456  -0.838  -1.906  -1.249  -1.456  -0.838  -1.906  -1.249  -1.456  -0.694  -0.138  -0.005  -0.039  -0.563  -0.586  -0.242  -0.067  0.653  1.324  0.568  0.495  1.148  0.619  0.944  1.095  0.760  0.859  0.813  0.707 | | 0.432  0.149  0.287  0.233  0.187  0.005  0.094  0.039  0.433  0.149  0.287  0.233  0.500  0.870  0.994  0.962  0.570  0.557  0.785  0.935  1.921  3.762  1.763  1.640  3.153  1.857  2.572  2.990  2.137  2.361  2.254  2.029 | | 0.049  0.295  0.031  0.140  0.049  0.296  0.028  0.135  0.049  0.296  0.030  0.127  0.043  0.843  0.992  0.948  0.047  0.715  0.490  0.919  0.050  0.726  0.191  0.481  0.035  0.770  0.186  0.350  0.018  0.558  0.062  0.250 | | 0.539  0.539  0.539  0.364  0.988  0.858  0.795  0.943 | | 0.734  0.734  0.734  0.710  0.167  0.260  0.402  0.285 |

Abbreviations: IVW-inverse variance weighting; MR-mendelian randomization; OR—odds ratio; PHN- postherpetic neuralgia.

**Supplementary Table 4** Reverse causal association between HZ and gut microbiota

| Exposure | Outcome | NO.of  SNP | Methods | *β* | *OR* | | *P* | | *P* of  Pleiotropy | | *P* of  Heterogeneity | |
| --- | --- | --- | --- | --- | --- | --- | --- | --- | --- | --- | --- | --- |
| HZ | class Deltaproteobacteria | 11 | IVW | -0.007 | | 0.992 | | 0.862 | | 0.865 | 0.064 |  |
|  |  | 11 | MR-Egger | -0.114 | | 0.886 | | 0.315 | |  |  |  |
|  |  | 11 | Weighted median | -0.051 | | 0.986 | | 0.785 | |  |  |  |
|  |  | 11 | Weighted mode | -0.066 | | 0.979 | | 0.756 | |  |  |  |
|  | order Desulfovibrionales | 11 | IVW | -0.010 | | 0.990 | | 0.825 | | 0.271 | 0.078 |  |
|  |  | 11 | MR-Egger | -0.129 | | 0.879 | | 0.272 | |  |  |  |
|  |  | 11 | Weighted median | -0.018 | | 0.983 | | 0.736 | |  |  |  |
|  | family Desulfovibrionaceae  genus Coprococcus2  phylum Cyanobacteria  genus Eubacterium rectale group | 11  11  11  11  11  11  11  11  11  11  11  11  11  11  11  11  11 | Weighted mode  IVW  MR-Egger  Weighted median  Weighted mode  IVW  MR-Egger  Weighted median  Weighted mode  IVW  MR-Egger  Weighted median  Weighted mode  IVW  MR-Egger  Weighted median  Weighted mode | -0.028  -0.010  -0.130  -0.018  -0.027  -0.065  -0.026  -0.078  -0.075  0.063  -0.038  0.074  0.076  0.064  0.126  0.059  0.093 | | 0.973  0.990  0.878  0.981  0.973  0.937  0.974  0.925  0.927  1.065  0.962  1.077  1.079  1.066  1.133  1.060  1.097 | | 0.687  0.815  0.265  0.714  0.682  0.109  0.817  0.174  0.337  0.207  0.768  0.242  0.433  0.044  0.155  0.170  0.163 | | 0.264  0.699  0.404  0.428 | 0.086  0.361  0.425  0.885 |  |

Abbreviations: HZ- herpes zoster; IVW-inverse variance weighting; MR-mendelian randomization; OR—odds ratio.

**Supplementary Table 5** Reverse causal association between PHN and gut microbiota

| Exposure | Outcome | NO.of  SNP | Methods | *β* | *OR* | | *P* | | *P* of  Pleiotropy | | *P* of  Heterogeneity | |
| --- | --- | --- | --- | --- | --- | --- | --- | --- | --- | --- | --- | --- |
| PHN | class Coriobacteriia  order Coriobacteriales  family Coriobacteriaceae  genus Lachnospiraceae NK4A136 group  genus Ruminococcaceae UCG011  genus Candidatus Soleaferrea  genus Eubacterium rectale group  genus Methanobrevibacter | 3  3  3  3  3  3  3  3  3  3  3  3  3  3  3  3  3  3  3  3  3  3  3  3  3  3  3  3  3  3  3  3 | IVW  MR-Egger  Weighted median  Weighted mode  IVW  MR-Egger  Weighted median  Weighted mode  IVW  MR-Egger  Weighted median  Weighted mode  IVW  MR-Egger  Weighted median  Weighted mode  IVW  MR-Egger  Weighted median  Weighted mode  IVW  MR-Egger  Weighted median  Weighted mode  IVW  MR-Egger  Weighted median  Weighted mode  IVW  MR-Egger  Weighted median  Weighted mode | -0.018  0.046  -0,016  -0.012  -0.018  0.046  -0.016  -0.011  -0.018  0.046  -0.016  -0.012  0.008  -0.038  0.002  0.000  0.029  0.170  0.044  0.051  0.016  -0.019  0.023  0.030  0.012  -0.041  0.016  0.024  0.101  0.403  0.065  0.047 | | 0.982  1.047  0.984  0.988  0.982  1.047  0.984  0.988  0.982  1.047  0.984  0.988  1.008  0.963  1.002  1.000  1.030  1.186  1.046  1.052  1.016  0.981  1.023  1.030  1.011  0.960  1.016  1.024  1.106  1.496  1.068  1.048 | | 0.257  0.605  0.432  0.676  0.049  0.257  0.440  0.694  0.257  0.605  0.432  0.703  0.612  0.665  0.913  0.985  0.405  0.446  0.278  0.425  0.552  0.892  0.492  0.506  0.470  0.640  0.441  0.452  0.123  0.293  0.237  0.557 | | 0.491  0.491  0.491  0.599  0.496  0.798  0.555  0.363 | 0.428  0.428  0.428  0.745  0.598  0.806  0.547  0.039 |  |

Abbreviations: IVW-inverse variance weighting; MR-mendelian randomization; OR-odds ratio; PHN- postherpetic neuralgia.
